# Role of multimeric analysis of von Willebrand factor (VWF) in von Willebrand disease (VWD) diagnosis: Lessons from the PCM-EVW-ES Spanish project

**DOI:** 10.1371/journal.pone.0197876

**Published:** 2018-06-20

**Authors:** Almudena Pérez-Rodríguez, Javier Batlle, Irene Corrales, Nina Borràs, Ángela Rodríguez-Trillo, Esther Lourés, Ana Rosa Cid, Santiago Bonanad, Noelia Cabrera, Andrés Moret, Rafael Parra, María Eva Mingot-Castellano, Nira Navarro, Carmen Altisent, Rocío Pérez-Montes, Shally Marcellini, Ana Moreto, Sonia Herrero, Inmaculada Soto, Nuria Fernández Mosteirín, Víctor Jiménez-Yuste, Nieves Alonso, Aurora de Andrés Jacob, Emilia Fontanes, Rosa Campos, María José Paloma, Nuria Bermejo, Rubén Berrueco, José Mateo, Karmele Arribalzaga, Pascual Marco, Ángeles Palomo, Nerea Castro Quismondo, Belén Iñigo, María del Mar Nieto, Rosa Vidal, María Paz Martínez, Reyes Aguinaco, Maria Tenorio, María Ferreiro, Javier García-Frade, Ana María Rodríguez-Huerta, Jorge Cuesta, Ramón Rodríguez-González, Faustino García-Candel, Manuela Dobón, Carlos Aguilar, Fernando Batlle, Francisco Vidal, María Fernanda López-Fernández

**Affiliations:** 1 Servicio Hematología, Complexo Hospitalario Universitario A Coruña, INIBIC, A Coruña, Spain; 2 Banc de Sang i Teixits, Barcelona, Spain; 3 Unitat d’Hemofilia, Vall d’Hebron Research Institute, Universitat Autònoma de Barcelona (VHIR-UAB), Barcelona, Spain; 4 Servicio Hematología, Hospital Universitario y Politécnico La Fe, Valencia, Spain; 5 Servicio Hematología, Hospital Universitari Vall d’Hebron, Barcelona, Spain; 6 Servicio Hematología, Hospital Regional Universitario de Málaga, Málaga, Spain; 7 Servicio Hematología, Hospital Universitario Dr. Negrín, Las Palmas de Gran Canaria, Spain; 8 Servicio Hematología, Hospital Universitario Marqués de Valdecilla, Santander, Spain; 9 Servicio Hematología, Salud Castilla y León, Segovia, Spain; 10 Servicio Hematología, Hospital Universitario Cruces, Barakaldo, Spain; 11 Servicio Hematología, Hospital Universitario de Guadalajara, Guadalajara, Spain; 12 Servicio Hematología, Hospital Universitario Central de Asturias, Oviedo, Spain; 13 Servicio Hematología, Hospital Universitario Miguel Servet, Zaragoza, Spain; 14 Servicio Hematología, Hospital Universitario La Paz, Madrid, Spain; 15 Servicio Hematología, Hospital Infanta Cristina, Badajoz, Spain; 16 Servicio Hematología, Complexo Hospitalario Universitario Santiago de Compostela, Spain; 17 Servicio Hematología, Hospital Universitario Lucus Augusti, Lugo, Spain; 18 Servicio Hematología, Hospital Jerez de la Frontera, Cádiz, Spain; 19 Servicio Hematología, Hospital Virgen del Camino, Pamplona, Spain; 20 Servicio Hematología, Hospital San Pedro de Alcántara, Cáceres, Spain; 21 Servicio Hematología, Hospital Sant Joan de Deu, Barcelona, Spain; 22 Servicio Hematología, Hospital Sta Creu i St Pau, Barcelona, Spain; 23 Servicio Hematología, Hospital Universitario Fundación Alcorcón, Madrid, Spain; 24 Servicio Hematología, Hospital General de Alicante, Alicante, Spain; 25 Servicio Hematología, Hospital Universitario 12 de Octubre, Madrid, Spain; 26 Servicio Hematología, Hospital Clínico San Carlos, Madrid, Spain; 27 Servicio Hematología, Complejo Hospitalario de Jaén, Jaén, Spain; 28 Servicio Hematología, Fundación Jiménez Díaz, Madrid, Spain; 29 Servicio Hematología, Hospital Nuestra Sra. de Sonsoles, Ávila, Spain; 30 Servicio Hematología, Hospital Joan XXIII, Tarragona, Spain; 31 Servicio Hematología, Hospital Ramón y Cajal, Madrid, Spain; 32 Servicio Hematología, Hospital Montecelo, Pontevedra, Spain; 33 Servicio Hematología, Hospital Río Hortega, Valladolid, Spain; 34 Servicio Hematología, Hospital Gregorio Marañón, Madrid, Spain; 35 Servicio Hematología, Hospital Virgen de la Salud, Toledo, Spain; 36 Servicio Hematología, Hospital Severo Ochoa, Madrid, Spain; 37 Servicio Hematología, Hospital Universitario Virgen Arrixaca, Murcia, Spain; 38 Servicio Hematología, Hospital Lozano Blesa, Zaragoza, Spain; 39 Servicio Hematología, Hospital Santa Bárbara, Soria, Spain; 40 Department of Research, Lapisoft Project S.L., A Coruña, Spain; 41 CIBER de Enfermedades Cardiovasculares (CIBERCV), Barcelona, Spain; Institut d'Investigacions Biomediques de Barcelona, SPAIN

## Abstract

The multimeric analysis (MA) of plasma von Willebrand factor (VWF) evaluates structural integrity and helps in the diagnosis of von Willebrand disease (VWD). This assay is a matter of controversy, being considered by some investigators cumbersome and only slightly informative. The centralised study ‘Molecular and Clinical Profile of von Willebrand Disease in Spain (PCM-EVW-ES)’ has been carried out by including the phenotypic assessment and the genetic analysis by next generation sequencing (NGS) of the VWF gene (VWF). The aim of the present study was to evaluate the role of MA to the diagnosis of these patients and their potential discrepancies. Two hundred and seventy out of 480 patients centrally diagnosed with VWD had normal multimers, 168 had abnormal multimers and 42 a total absence of multimers. VWF MA was of great significance in the diagnosis of 83 patients (17.3%), it was also of help in the diagnosis achieved in 365 additional patients (76%) and was not informative in 32 cases (6.7%). With regard to discrepancies, 110 out of 480 (23%) patients centrally diagnosed with VWD presented some kind of discordance between VWF:RCo/VWF:Ag and/or VWF:CB/VWF:Ag ratios, multimeric study and/or genetic results. The VWF MA was key in the presence of novel mutations as well as in cases with phenotypic discrepancies. A comparison between the contribution of MA and VWF:CB showed a clearly higher contribution of the former in the diagnostic process. These data seem to reinforce the relevance of the VWF MA in VWD diagnosis, despite all its limitations.

## Introduction

von Willebrand disease (VWD) is the most frequent inherited bleeding disorder caused by defects in the amount, structure or function of von Willebrand factor (VWF), which has an important role in primary haemostasis, as well as to bind and stabilize coagulation factor VIII (FVIII) **[1,2]**

Accurate diagnosis and classification of VWD are essential for optimal management and genetic counselling, but frequently are difficult due to the variability of its clinical expression, and difficulties in standardising a panel of diagnostic tests, some of which are not available in all laboratories **[3–11]**.

The screening tests used to classify VWD are the VWF antigen (VWF:Ag), VWF ristocetin cofactor activity (VWF:RCo), factor VIII activity (FVIII:C), and VWF collagen binding (VWF:CB), whereas the confirmatory tests include ristocetin-induced platelet agglutination (RIPA), VWF capacity to bind exogenous FVIII (VWF:FVIIIB), VWF propeptide (VWFpp), and multimeric analysis (MA), which are especially useful to differentiate between the different types of VWD.

The latest official classification of VWD refers to the VWF multimeric profile **[12]**, considering that the MA is in the phenotypic classification an integral part of the diagnostic process. Moreover, the diagnosis and classification of VWD is based on phenotype and not on genotype because it does not even require the presence of a genetic mutation in the VWF gene (*VWF*).

However, the role of the VWF MA in VWD diagnosis has raised some controversy. MA is a cumbersome methodology indeed, time consuming and requiring quite considerable experience. Also, even in the experienced laboratories there is a high error rate, with inconsistent interpretation of profiles and false classification **[13]**. Therefore, some laboratories have now dispensed with MA technique, claiming that VWD classification can be reliably achieved with a combination of FVIII:C, some standard VWF assays (VWF:Ag, VWV:RCo and VWF:CB), as well as genotype analysis in some cases **[14,15]**. From a clinician's perspective, some opinion considers that what really matters in VWD diagnosis is whether a patient responds to desmopressin (DDAVP), and formal VWD subclassification in a laboratory is thus not necessary.

Despite this opinion, when MA is performed with high quality it may predict the presence of *VWF* mutations **[16]**. It allows to determine whether there is a loss of high molecular weight multimers (HMWM) to help classifying patients with type 2 VWD, and to detect more minor abnormalities in the VWF satellite banding pattern **[16,17]**.

The need for molecular and clinical characterisation of VWD in Spain prompted the creation of a multicentre project (Molecular and Clinical Profile of von Willebrand Disease in Spain; PCM-EVW-ES) that resulted in the one of the largest prospective cohort study of patients with all types of VWD **[3,18]**. In this project, the role of next generation sequencing (NGS) in VWD diagnosis was evaluated demonstrating it can resolve many of the drawbacks and limitations of phenotyping **[19].**

In this regard, taking the advantage of the wide VWD cohort of patients enrolled and finely defined in the PCM-EVW-ES Project, assuming the knowledge of their final diagnosis, the present study was focused to the investigation of the global contribution (role) and limitations of the MA in the VWD diagnostic process.

## Material and methods

### Study design

The ‘Molecular and Clinical Profile of von Willebrand Disease in Spain (PCM–EVW–ES)’ project is a survey that centrally analysed a large cohort of patients from Spain. For further details of the study design and recruitment see references **[3,18].** The study recruited 556 patients, of whom 480 were finally diagnosed with VWD, from 38 centres nationwide. The revised International Society on Thrombosis and Haemostasis (ISTH) classification for diagnosis was used **[12]** although the inclusion of some new categories, suggested by several investigators, were made (**S1 Table).**

### Ethics statement

This study was performed according to the guidelines of the Declaration of Helsinki, and it was approved by the Internal Review Board (IRB) from the Instituto de Investigations Biomedical of A Corunna (INIBIC) and from the Ethics Committee of Clinical Research of Galicia, Spain (CEIC), with the Registration Code Galicia CEIC: 2009/299). All participants provided written informed consent. In case of minors, we obtained consent from parents or guardians. Copies of the IRB statement and the ETHIC Committee indicating all the members of this Committee that were involved are provided with the present manuscript.

All data were fully anonymized before we accessed them and/or whether the IRB or ethics committee waived the requirement for informed consent. The study fully complies with the Spanish Protection Data Law regulation (LOPD).

### Patients and controls

Patients of any age previously diagnosed at the local centers with VWD between November 2010 and 2013 were recruited. All patients had to fulfill one or more of the following inclusion criteria: 1) VWF:Ag, VWF:RCo and/or VWF:CB ≤ 30 IU/dL (%), observed on two or more occasions; 2) detection of multimeric abnormalities; 3) in case of isolated FVIII deficiency it will be necessary to provide demonstration of a decreased VWF:FVIIIB; 4) presence of one or more *VWF* mutations; 5) presence of RIPA at a low ristocetin concentration. The exclusion criteria were the presence of any data suggesting acquired von Willebrand syndrome (AVWS). A 30 IU/dL VWF cut-off plasma level was used when a low VWF level was the only fulfilled criteria **[3,18]**. The main demographic data of the participants are included in Table 1.

**Table 1:** Demographic data of the cohort of patients.

Demographic data	
**Gender**	**N patients**
Male	213
Female	267
**Age (years), range**	**N patients**
Male	38,18 (4–90)
Female	38,56 (3–92)
**Blood group**	**N patients**
Group A	233 (48,4%)
Group B	41 (8,6%)
Group AB	36 (7,5%)
Group O	156 (32,5%)
NA	14 (2,9%)

NA: Not available

As controls, 30 healthy subjects unrelated to any of the studied patients were also recruited for phenotypic analysis. Blood and plasma were collected and sent to three central laboratories designated for the project.

### Phenotypic analysis

It was carried out as indicated in our previous publication **[18]**. Basically FVIII:C, VWF:Ag, VWF:RCo and VWF:CB were measured in all recruited individuals’ plasma samples. FVIII:C was assessed by a one-stage clotting assay using Actin FS as activator on a Behring Coagulation Timer (BCT) (Siemens Healthcare Diagnostics). The VWF:Ag was measured using the ELISA kit, DG–EIA VWF (Diagnostic Grifols). VWF:CB with collagen type III was determined by the ELISA kit DG–CBA VWF (Diagnostic Grifols), on a Triturus Immunoassay System. In some samples VWF:CB was assayed by using type VI collagen (TECHNOZYMR VWF:CBA ELISA Collagen Type VI, Technoclone). The VWF:RCo test was performed on a BCT using the BC von Willebrand Reagent (Siemens Healthcare Diagnostics), and also by using conventional light transmission aggregometry. Throughout literature, abnormal VWF:RCo/VWF:Ag and VWF:CB/VWF:Ag has been defined between 0.5–0.7 to distinguish between VWD type 1 and type 2 **[12].** In our study, we considered a ratio cut off 0.7 as indicative of a qualitative VWF defect.

The VWF:FVIIIB was assessed using an in-house ELISA **[20]** in all patients who presented with a FVIII:C/VWF:Ag ratio <1, and in those in whom a type 2N mutation was found.

Lyophilized, reconstituted normal control plasma calibrated to the World Health Organization standard was used as reference.

### VWF multimeric analysis

VWF multimeric distribution was analysed in all 556 patients and in the healthy controls.

This assay is based on the method originally described by Ruggeri and Zimmerman in 1981 **[21]** and modified by our research team **[22].** Samples were diluted in a loading buffer according to the amount of antigen and subjected to non-reducing electrophoresis in the presence of SDS denaturing agent in low-resolution (1% Seakem HGT agarose; Lonza, Rockland ME, USA) and high resolution (2% agarose type VII; Sigma, St Louis, USA) gels.

Afterwards, the proteins were transferred by electroblotting (Hoeffer TE62, Amersham Bioscience) to an immobilon polyvinylidene difluoride (PVDF) membrane (Millipore Corporation, Bedford, MA, USA), previously treated with methanol and distilled water. All incubation and washing steps were performed in low-fat milk. Visualisation of VWF multimers was achieved by incubating the membranes with a rabbit anti-human VWF antibody (Polyclonal Rabbit Anti-Human Von Willebrand Factor, Dako, Glostrup, Denmark), followed by a goat anti-rabbit IgG (Polyclonal Goat Anti-Rabbit Immunoglobulins/Biotinylated, Dako, Glostrup, Denmark) and finally with an anti-goat IgG that contains alkaline phosphatase (Extravidin(TM)-Alkaline Phosphatase; Sigma,St. Louis, USA). The multimeric profile of the samples was visualized using a histological staining for the enzyme and chromogenic substrates (Fast Blue RR SALT, Sigma-aldrich, St Louis, USA). After staining the membranes were scanned using the ImageQuant program (General Dynamics), obtaining the densitometric image and the quantification of the area under the curve of each peak or band **[23].** VWF multimers of patients’ plasma were classified as loss of HMWM (↓HMWM) or normal multimers by comparison with the reference plasma (pool of 30 HC). In some samples there was no clear separation between individual oligomer triplets, leading to a smeary multimer pattern, these samples were designated as ‘*smear*’ **[16]**.

### Genetic analysis

Mutations in the *VWF* were analysed in the 556 individuals by NGS as described previously **[18,19,24].**

## Results

Five hundred and fifty-six patients from 330 families historically diagnosed with VWD by their local centres were recruited into the project. After central phenotypic studies, VWD was confirmed in 442 patients; however, after genetic analysis 480 patients fulfilled the recruitment criteria of this study.

Regarding the multimeric profiles, 270 patients were normal, 168 abnormal and 42 with a total absence of multimers. The distribution of the patients according to their MA and type of VWD diagnosis is shown in **S1 Fig**. It is important to emphasise that 14 patients whose MA was difficult to define (14 type 2A), were first classified as ‘result not conclusive’, but due to the similarity with the normal multimeric pattern those individuals were finally considered patients with normal MA **(S2 Fig)**.

### Contribution of MA in the diagnosis of VWD

To evaluate the contribution of MA in the entire analytical process, we analysed this cohort of patients under consideration of four distinct successive steps according to the laboratory tests assessment, compared to the final diagnosis achieved in this project:

- Screening test considering only FVIII:C, VWF:Ag, VWF:RCo and VWF:CB.
- Confirmatory 1, including VWF:FVIIIB values.
- Confirmatory 2, adding MA.
- Molecular study, including the genetic analysis.

Due to the limited number of local centres providing RIPA, this parameter was not considered in this evaluation.

Regarding the role of MA, three different categories were established: 1) Great significance: MA was necessary to establish a clear diagnosis; 2) Concordant: once established a clear diagnosis with other tests, the MA agreed with such diagnosis; 3) Not informative: the MA did not provide information for the diagnosis.

**Fig 1:**
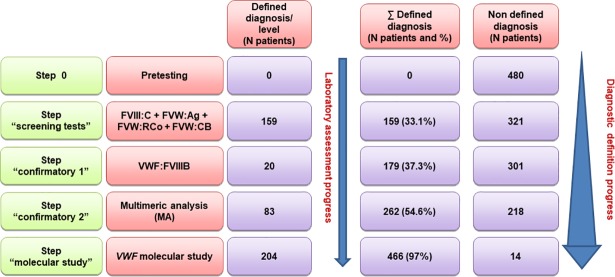
Evaluation of the contribution of different laboratory steps in the diagnosis definition. Four different successive laboratory assessment steps are considered. The progress in the diagnostic definition of the patients according to each step is shown. After the first and second steps, the multimeric analysis (MA) was of great significance in the diagnosis definition of 83 additional patients. Moreover, MA was in agreement with the diagnostic definition accomplished in steps 1 and 2 (179 patients). Finally, MA was also in agreement with the diagnostic definition achieved by molecular analysis in 186 additional patients. Thus, MA contributed to the diagnosis definition in a total of 448 (93.3%) patients.

**Fig 1** shows the result of this evaluation, and the progress regarding a clear diagnosis definition.

After the first step a defined phenotypic diagnosis orientation was concordant with the final diagnosis in 159 (33.1%) cases. In the second step, by including the VWF:FVIIIB test in those patients having a selective deficiency of FVIII:C or a clearly decreased FVIII:C/VWF:Ag ratio, the phenotypic analysis was concordant with the final diagnosis in 20 additional patients (37.3%). In the remaining patients, the MA was of great importance in the clarification of the phenotypic diagnosis in 83 new cases, being of help and concordant with the diagnosis achieved in the previous step in all the 179 patients (54.6%). After MA, the diagnosis of 218 cases remained not yet defined.

In the last group, the molecular study allowed a diagnostic definition in 204 additional patients, being the coincidence with the final diagnosis of 97%. However, it is important to emphasise that at this step, the MA agreed with molecular analysis results in 186 patients.

Also, it should be stressed that MA was not informative or showed some inconsistency in 32 cases. After the four steps, 14 cases remained not defined (2.92%).

In consequence, MA was globally of help (of great significance + concordant or in agreement) in the entire diagnostic process of 448 patients (93.3%).

### Contribution of MA in comparison to VWF:CB

**Fig 2:**
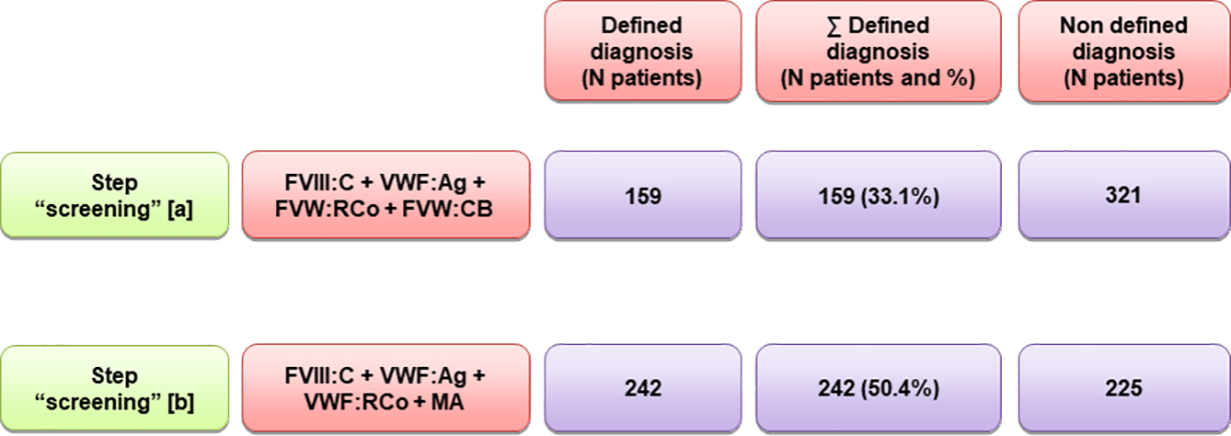
**Comparison between the diagnostic definition contribution of VWF:CB (in step “screening tests” [a]) and the multimeric analysis (MA) instead of VWF:CB first step, (step “screening tests” [b]).** A greater degree of efficiency was observed for MA (50.4% *versus* 33.1% for VWF:CB).

An additional comparison exercise was made between the step “screening tests” [a] (that includes VWF:CB assessment) and the contribution of MA substituting VWF:CB (step “screening tests” [b]) **(Fig 2).** The data showed that the MA (50.4%) was clearly more efficient than the VWF:CB (33.1%).

### Correlations MA-VWF:RCo/VWF:Ag-VWF:CB/VWF:Ag

With regards to discrepancies, 110 out of 480 (23%) patients centrally diagnosed with VWD presented some kind of discordance between VWF:RCo/VWF:Ag, VWF:CB/VWF:Ag, multimeric study and/or genetic analysis.

**Fig 3:**
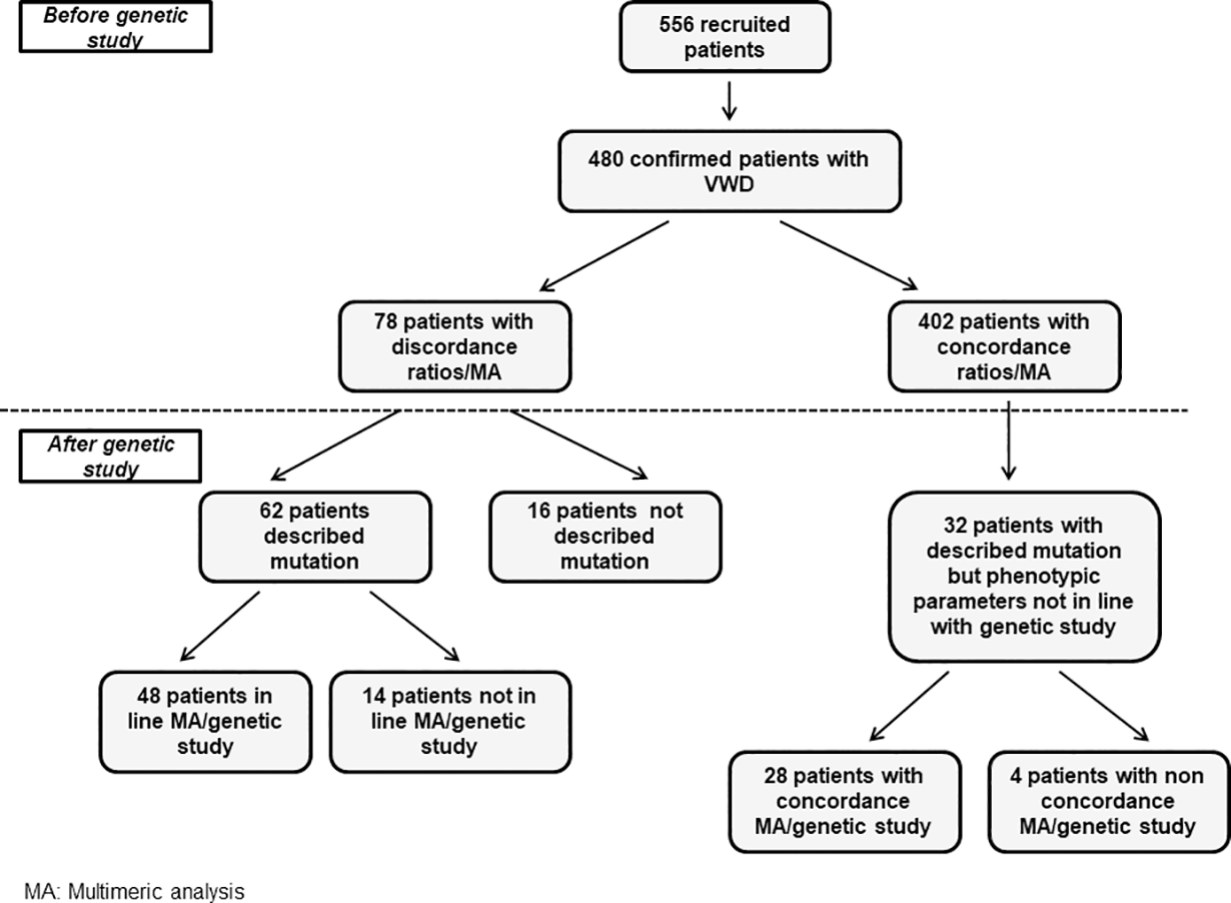
Distribution of patients according to their coincidence between ratios and multimeric analysis before genetic study and between multimeric analysis and mutation after genetic study. Of 110 patients with some type of discrepancy, in 76 (48 + 28) the MA was in line with the molecular study while in 18 (14+4) patients there was not concordance. In the remaining 16, the similarity could not be demonstrated because the mutation found has not been described previously.

Prior to the genetic study, 78 (16.2%) out of 480 patients centrally diagnosed with VWD presented discordance between MA and VWF:RCo/VWF:Ag and/or VWF:CB/VWF:Ag, and in 66 of them MA was of help for a correct classification. The remaining 402 (83.8%) ratios and MA were in line (Table 2 and Fig 3). In three out of 78 patients the MA was discordant respect to VWF:RCo/VWF:Ag, in 49 with the VWF:CB/VWF:Ag ratio and in the remaining 26 patients with both ratios.

**Table 2:** Patients with discordance between VWF:RCo/VWF:Ag-VWF:CB/VWF:Ag-multimeric pattern.

Patient	FVIII:C(IU/dL)	VWF:Ag (IU/dL)	VWF:Rco (IU/dL)	VWF:CB (IU/dL)	VWF:RCo/ VWF:Ag	VWF:CB/ VWF:Ag	Multimeric analysis	Mutation	Type
C02P026F08	44	21	7.4	8.8	0.35	0.42	*Smear*	p.Arg1315Cys*	2A/2M
C02P027F08	28	11	7.1	6.4	0.64	0.58	*Smear*	p.Arg1315Cys*	2A/2M
C02P034F12	23	26	8.4	14	0.32	0.54	*Smear*	p.Arg1315Cys*	2A/2M
C03P017F76	39	44	11.8	23	0.27	0.52	*Smear*	p.Arg1374Cys*	2A/2M
C13P009F07	20	9.5	5	5.8	0.53	0.61	*Smear*	p.Arg1315Cys*	2A/2M
C13P016F07	21	11	5	6.7	0.45	0.61	*Smear*	p.Arg1315Cys*	2A/2M
C01P038F20	20	20	5.3	13	0.27	0.65	*Smear*	p.Arg1315Cys*	2A/2M
C01P002F02	34	16	4.4	9.2	0.28	0.58	*Smear*	p.Arg1374Cys*	2A/2M
C01P003F02	45	20	6.7	13	0.34	0.65	*Smear*	p.Arg1374Cys*	2A/2M
C01P020F02	47	32	11	19	0.34	0.59	*Smear*	p.Arg1374Cys*	2A/2M
C01P022F02	59	54	18	30	0.33	0.55	*Smear*	p.Arg1374Cys*	2A/2M
C01P039F02	24	23	12	15	0.52	0.65	*Smear*	p.Arg1374Cys*	2A/2M
C01P007F04	38	25	7.8	15	0.31	0.6	*Smear*	p.Arg1374Cys*	2A/2M
C01P015F04	36	19	7.8	11	0.41	0.58	*Smear*	p.Arg1374Cys*	2A/2M
C01P023F04	27	30	13	18	0.43	0.6	*Smear*	p.Arg1374Cys*	2A/2M
C01P033F04	32	27	10	16	0.37	0.59	*Smear*	p.Arg1374Cys*	2A/2M
C01P042F16	26	27	4.9	17	0.18	0.63	*Smear*	p.Arg1374Cys*	2A/2M
C01P071F16	17	13	6.6	6.7	0.51	0.51	*Smear*	p.Arg1374Cys*	2A/2M
C37P003F03	44	30	25	20	0.83	0.66	*Smear*	p.Cys2491Arg	2A/2M
C30P012F07	18	32	28	19	0.88	0.59	*Smear*	p.Arg763Ser	2A/2M y 2N
C30P013F08	53	78	31.8	34	0.41	0.43	*Smear*	p.Arg763Ser	2A/2M y 2N
C02P047F18	30	9.3	6.4	6.1	0.69	0.66	*Smear*	p.Pro1824His*	1 Smeary
C02P001F01	19	9.7	2.6	6	0.27	0.62	Normal	p.Arg1205His*	1
C13P006F04	16	10	6	6.1	0.6	0.61	Normal	p.Arg1205His*	1
C32P004F04	6.3	9.8	4	5	0.41	0.51	Normal	c.2821-123A>C	1
C37P005F05	44	26	18	18	0.69	0.69	Normal	p.Arg960Trp*	1
C39P019F08	30	23	12	16	0.52	0.69	Normal	c.3390C>T*	1
C32P007F07	46	69	35	34	0.51	0.49	Normal	p.Ile482Met	1H
C30P004F03	26	16	5.4	13	0.34	0.81	*↓HMWM*	p.Leu1307Pro*	2A
C30P007F03	24	13	5.9	9.9	0.45	0.76	*↓HMWM*	p.Leu1307Pro*	2A
C30P010F03	21	11	5.6	9.2	0.51	0.83	*↓HMWM*	p.Leu1307Pro*	2A
C03P007F04	30	18	6.4	15	0.36	0.83	↓HMWM	p.Arg1374His*	2A
C03P008F04	26	14	7	14	0.5	1	↓HMWM	p.Arg1374His*	2A
C30P005F04	26	22	5.8	15	0.26	0.68	Normal	p.Arg1374His	2A
C30P015F04	29	31	5	14	0.16	0.45	Normal	p.Arg1374His	2A
C30P016F04	26	22	5	12	0.23	0.54	Normal	p.Arg1374His	2A
C30P019F04	21	26	5	12	0.19	0.46	Normal	p.Arg1374His	2A
C30P020F04	45	62	5.6	27	0.09	0.43	Normal	p.Arg1374His	2A
C30P021F04	33	35	5.8	17	0.19	0.49	Normal	p.Arg1374His	2A
C30P022F04	25	30	5.6	14	0.19	0.47	Normal	p.Arg1374His	2A
C30P024F04	19	25	5.4	14	0.22	0.56	Normal	p.Arg1374His	2A
C30P025F04	25	26	4	11	0.15	0.42	Normal	p.Arg1374His	2A
C30P026F04	27	33	4	18	0.12	0.55	Normal	p.Arg1374His	2A
C30P031F04	21	22	4	11	0.18	0.5	Normal	p.Arg1374His	2A
C30P032F04	41	57	9	29	0.16	0.51	Normal	p.Arg1374His	2A
C30P034F04	21	21	5	9.8	0.24	0.47	Normal	p.Arg1374His	2A
C30P036F04	30	23	5	11	0.22	0.48	Normal	p.Arg1374His	2A
C39P005F03	26	13	7.1	14	0.55	1.08	↓HMWM	p.Arg1374His*	2A
C39P006F03	23	12	4.7	14	0.39	1.17	↓HMWM	p.Arg1374His*	2A
C06P021F15	31	13	5.7	13	0.44	1	↓HMWM	p.Arg1374His*	2A
C06P022F15	30	16	6.1	16	0.38	1	↓HMWM	p.Arg1374His*	2A
C39P001F02	35	18	5	17	0.28	0.94	↓HMWM	p.Arg1374His*	2A
C39P004F02	29	13	6.7	15	0.52	1.15	↓HMWM	p.Arg1374His*	2A
C39P011F06	31	17	7.7	21	0.45	1.23	↓HMWM	p.Ser1506Leu*	2A
C39P012F06	13	8.2	5	6.9	0.61	0.84	↓HMWM	p.Ser1506Leu*	2A
C42P010F10	52	69	58.5	45	0.85	0.65	↓HMWM	p.Arg1597Gln*	2A
C22P005F05	47	12	6.9	11	0.58	0.92	↓HMWM	p.Val1414Gly	2A
C07P006F02	26	17	4	12	0.24	0.71	↓HMWM	p.Asp1614Asn	2A
C02P078F07	55	34	24	21	0.71	0.62	↓HMWM	p.Arg1308Cys*	2B
C27P015F08	63	33	18.5	25	0.56	0.76	↓HMWM	p.Arg1306Trp*	2B
C14P001F01	15	9	6	7.6	0.67	0.84	↓HMWM	p.Arg1306Trp*	2B
C12P015F08	48	56	45	42	0.8	0.75	↓HMWM	p.Arg1306Gln*	2B
C35P009F04	24	23	18	16	0.78	0.69	↓HMWM	p.Arg1308Cys*	2B
C01P066F32	26	16	4	11	0.25	0.69	Normal	p.Gly1415Asp*	2M
C12P020F13	67	84	55	53	0.65	0.63	Normal	p.Arg1399His*	2M
C27P010F06	18	6.8	4	4.3	0.59	0.63	Normal	p.Leu1382Pro*	2M
C03P023F32	21	12	4.4	7.7	0.37	0.64	Normal	p.Val1409Phe	2M
C03P024F32	26	25	7.2	12	0.29	0.48	Normal	p.Val1409Phe/p.Arg1399His	2M
C03P025F32	65	109	17	42	0.16	0.38	Normal	p.Val1409Phe	2M
C12P023F16	213	272	186	178	0.68	0.65	Normal	p.Arg1395Trp	2M
C30P027F10	34	36	16	20	0.44	0.55	Normal	p.Arg1779Leu	2M
C30P028F10	19	28	13.6	16	0.49	0.57	Normal	p.Arg1779Leu	2M
C44P003F02	11	16	5	11	0.31	0.69	Normal	p.Gly1415Asp/Arg854Gln*	2M
C44P008F06	23	28	6.2	19	0.22	0.68	Normal	p.Val1409Phe	2M
C44P009F06	60	46	10.4	32	0.23	0.69	Normal	p.Val1409Phe	2M
C44P010F06	33	33	7.4	21	0.22	0.64	Normal	p.Val1409Phe	2M
C03P022F25	85	88	46	48	0.52	0.55	Normal	c.7082-2A>G/c.7730-177G>T*	3 carrier
C32P011F10	88	51	23.5	21	0.46	0.41	Normal	p.Gln1311Ter*	3 carrier
NV	60–140	47–190	50–170	60–130	>0.7	>0.7	–	–	–

NV: Normal value; FVIII:C: procoagulant factor VIII; VWF:Ag: VWF antigen; VWF:RCo: VWF ristocetin cofactor activity; VWF:CB: VWF collagen binding; ↓HMWM: decreased proportion of high molecular weight multimers.

Mutations previously described are indicated in bold type.

* Multimeric pattern consistent with the mutation.

### Correlations phenotypic/genotypic study

Once the genetic study by NGS was performed, it was observed that 62 (79.5%) out of 78 patients with discordance ratios-MA carried a previously described mutation **(Table 2)**. In 48 of them the multimeric pattern was consistent with the detected mutation, whereas in the remaining 14 the multimeric structure was not in line with the genetic study **(Fig 3 and Table 2)**. In these cases, the molecular study was very important to establish the diagnosis. Curiously, these 14 patients carried the previously described p.Arg1374His mutation **(S2 Fig)**. The remaining 16 patients out of 78 had novel mutations.

On the other hand, after mutation analysis it could be observed that in 32 out of 402 patients in whom the ratios and multimeric structure matched, there was no correlation with the genetic study **(Table 3)**. In 28 of them the multimeric pattern was consistent with the detected mutation, whereas in the remaining four it was not. **(Fig 3 and Table 3).**

**Table 3:** Patients with consistency between VWF:RCo/VWF:Ag-VWF:CB/VWF:Ag-multimeric pattern but not with genetic analysis.

Patient	FVIII:C (IU/dL)	VWF:Ag (IU/dL)	VWF:RCo (IU/dL)	VWF:CB (IU/dL)	VWF:RCo/ VWF:Ag	VWF:CB/ VWF:Ag	Type#	Multimeric analysis	Mutation	Type##
C02P049F18	37	11	6.6	8	0.6	0.73	2A/2M	*Smear*	p.Pro1824His*	1 Smeary
C02P050F18	37	10	6.9	7.1	0.69	0.71	2A/2M	*Smear*	p.Pro1824His*	1 Smeary
C02P016F01	34	16	9	16	0.56	1	2M	Normal	p.Arg1205His*	1
C13P012F09	20	14	9	11	0.64	0.79	2M	Normal	p.Arg1205His*	1
C14P003F03	61	28	20	18	0.71	0.64	2M	Normal	p.Gly160Arg*	1
C18P001F01	17	10	6.1	8.2	0.61	0.82	2M	Normal	p.Arg1205His/p.Val2330Gly*	1
C18P002F01	16	12	7	9.5	0.58	0.79	2M	Normal	p.Arg1205His/p.Val2330Gly*	1
C36P008F06	37	34	14	25	0.41	0.73	2M	Normal	p.Arg924Gln/c.3390C>T(p. =)*	1
C38P010F05	86	43	27.5	36	0.64	0.84	2M	Normal	p.Arg960Trp*	1
C39P017F08	4	26	17	19	0.65	0.73	2M	Normal	c.3390C>T*	1
C42P003F03	50	60	48	41	0.8	0.68	2M	Normal	p.Tyr1584Cys/p.Arg1916Gln*	1
C45P001F01	16	11	6.6	9.4	0.6	0.85	2M	Normal	p.Arg1205His*	1
C12P018F11	51	63	49	42	0.78	0.67	2M	Normal	p.Arg1583Trp*	1H
C19P006F04	74	46	38	31	0.83	0.67	2M	Normal	p.Gln2470Ter*	1H
C21P019F17	59	57	50	39	0.88	0.68	2M	Normal	p.Arg924Gln*	1H
C29P004F03	42	59	48	41	0.81	0.66	2M	Normal	p.Val1760Ile*	1H
C39P018F08	69	53	43	35	0.81	0.66	2M	Normal	c.3390C>T(p. =)*	1H
C02P071F27	67	27	23.6	36	0.87	1.33	1	Normal	p.Arg976Cys/p.Pro2063Ser	2A
C02P072F27	114	45	46	57	1.02	1.27	1	Normal	p.Arg976Cys/p.Pro2063Ser	2A
C30P011F04	26	18	5.8	15	0.32	0.83	2M	Normal	p.Arg1374His	2A
C30P029F04	19	19	5.7	13	0.3	0.72	2M	Normal	p.Arg1374His	2A
C01P036F18	17	16	24	20	1.5	1.25	1	Normal	p.Arg1399His*	2M
C05P013F12	78	50	47.6	39	0.95	0.78	1	Normal	p.Arg1399His*	2M
C07P007F05	97	32	39	24	1.22	0.75	1	Normal	p.Arg1399His/p.Cys2283Arg*	2M
C27P009F06	15	5.4	4	5.6	0.74	1.03	1	Normal	p.Leu1382Pro*	2M
C27P022F12	65	38	35.5	41	0.93	1.08	1	Normal	p.Ser1731Thr*	2M
C01P060F24	100	143	114	98	0.8	0.68	2M	Normal	p.Arg324Ter*	3 carrier
C03P038F127	82	67	37	56	0.55	0.84	2M	Normal	p.Gln1311Ter*	3 carrier
C03P039F127	68	66	34	47	0.52	0.71	2M	Normal	p.Gln1311Ter*	3 carrier
C27P041F21	66	42	25	48	0.59	1.14	2M	Normal	p.Tyr126Thrfster49*	3 carrier
C34P001F01	48	41	35	28	0.85	0.68	2M	Normal	p.Gly142Asp*	3 carrier
C39P010F05	128	104	92	57	0.88	0.55	2M	Normal	p.Gln2783Ter*	3 carrier
NV	60–140	47–190	50–170	60–130	>0.7	>0.7	–	–	–	–

NV: Normal value; FVIII:C: procoagulant factor VIII; VWF:Ag: VWF antigen; VWF:RCo: VWF ristocetin cofactor activity; VWF:CB: VWF collagen binding.

Mutations previously described are indicated in bold type.

* Multimeric pattern consistent with the mutation.

# Classification before genetic study.

## Classification after genetic study.

### Consistency between phenotypic and genotypic study according to types of VWD

#### Type 1 VWD

One hundred and fifty-nine patients were diagnosed with type 1 VWD in the PCM-EVW-ES project. Twenty-four of them presented some discordance at the time of the diagnosis **(S2 Table)**.

Prior to the genetic analysis, seven patients presented discordance between VWF:RCo/VWF:Ag and VWF:CB/VWF:Ag ratios and MA. All of them had values for both ratios less than 0.7 characteristic for type 2A or 2B VWD but with MA normal except patient C02P047F18 whose MA was type ‘smear’ **(S3 Fig)(S2 Table).**

After taking part in the genetic study, it could be observed that this last patient carried the mutation p.Pro1824His, with a historically controversial classification **[25, 26]**. Of the remaining six patients, two carried a novel mutation and the other four mutations previously known as causing type 1 VWD, being concordant in all of them the MA and the genotype.

Additionally, after *VWF* sequencing it was possible to verify that in 15 patients whose ratios and multimeric structure were matched, there was no concordance of results with the genetic study. All of them presented with a mutation previously described as responsible for type 1 VWD, however, considering only the phenotypic study they had been diagnosed as type 2M **(S3 Fig)**. For this reason, in the present study these patients were considered as type 1 VWD.

#### Type 2A VWD

**Fig 4:**
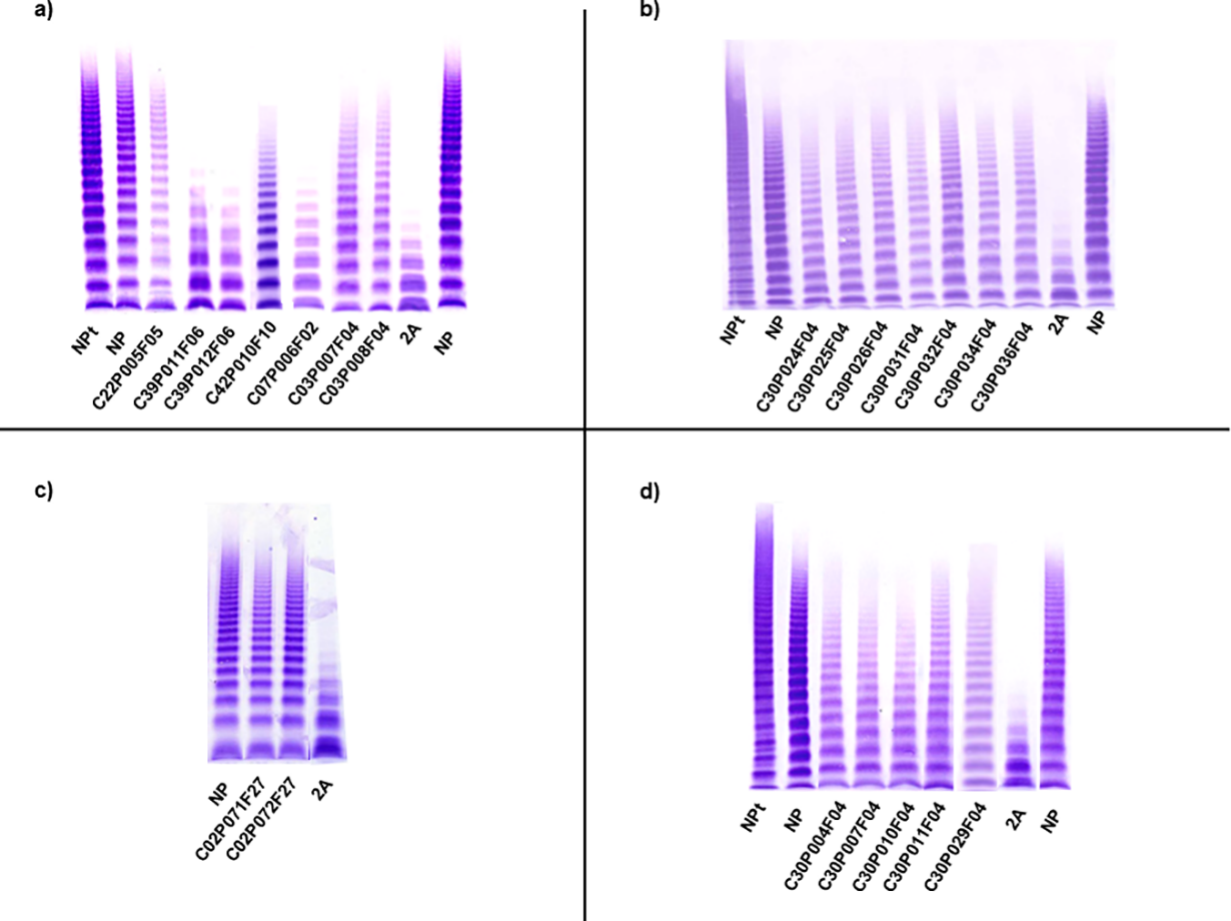
Multimeric analysis of von Willebrand factor (VWF) in low-resolution SDS-agarose gels in patients with type 2A VWD and some discrepancy. VWF from platelet lysate (NPt), plasmas of a normal subject (NP), patients with type 2A VWD and a patient with VWD type 2A (IIA) used as a control 2A are shown. **(a-b):** Patients with discrepancy between ratios and multimeric analysis; **(c-d):** Patients re-classified as type 2A on the basis of the genetic study.

A total of 111 patients with type 2A VWD were diagnosed in this project. Before carrying out the genetic study, 30 patients (27%) presented discrepancies between ratios and MA **(S3 Table).** Sixteen out of 30 had absence of HMWM, however, VWF:RCo/VWF:Ag and/or VWF:CB/VWF:Ag were higher than 0.7 **(Fig 4)**. On the other hand, in the remaining 14 patients, the MA was normal/almost normal, but both ratios were less than 0.7. These 14 patients belonged to the same family and they carried the p.Arg1374His mutation **(S3 Table) (S2 Fig and Fig 4).**

Once the genetic study results were obtained, it was confirmed that 28 out of 30 patients had a mutation previously described as causing type 2A VWD. In addition, surprisingly, it was observed that four new patients carried a known type 2A mutation but, according to their phenotypic study they had been previously classified as: type 1 (two patients: C02P071F27 and C02P072F27) and type 2M (two patients: C30P011F04 and C30P029F04)**(S3 Table)(Fig 4)**.

#### Type 2B VWD

Five out of 35 patients (14.3%) with type 2B VWD in this registry presented discordance between ratios and MA. In all of them the multimer pattern was characterised by a relative decrease of the large multimer. However, two patients had a VWF:RCo/VWF:Ag>0.7, two patients presented with a VWF:CB/VWF:Ag > 0.7 and finally, in one patient both ratios were higher than 0.7 **(S4 Table; S4 Fig).** The genetic analysis confirmed than the five patients had a known type 2B VWD mutation, showing concordance with the MA results but not with the VWF ratios.

#### Type 2M VWD

Prior to the genetic study, 13 out of 39 patients (33.3%) who were finally classified as type 2M VWD presented discrepancy between ratios and MA. All of them had normal MA pattern results, typical of type 2M VWD; however, both ratios were less than 0.7, typical of type 2A or type 2B VWD. After sequencing *VWF*, it could be observed that only five out of 13 were carrying mutations previously described **(S5 Table).** Two of these patients presented the p.Arg1399His mutation classified as type 2M VWD **[27]**, one other patient presented the p.Leu1382Pro mutation and the remaining two patients carried the p.Gly1415Asp mutation previously described as causing type 1 VWD **[28]** but considered in our project, in the light of phenotypic results, as type 2M VWD **(S5 Table)(S5 Fig)**. The other eight patients out of 13 did not have known mutations but they were considered to be type 2M VWD due to the fact that they presented with normal MA and decreased VWF:RCo/VWF:Ag ratio. In the 13 cases, the multimeric study was of great significance to establish a correct classification as type 2M VWD.

Interestingly, the genetic study allowed the identification of five new patients with type 2M VWD who, according to the phenotypic study, had been diagnosed as type 1 VWD.

Three of them carried the p.Arg1399His mutation which affected the binding to type VI collagen, one had the p.Ser1731Thr mutation (affecting the binding to type III collagen) and the other the p.Leu1382Pro mutation (impairing a type of collagen not specified)**(S5 Table).**

In all patients with previously described mutations the MA always matched with the genetic result.

#### Type 2A/2M VWD

**Fig 5:**
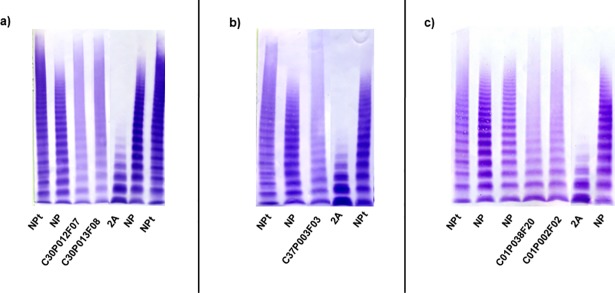
Multimeric analysis of von Willebrand factor (VWF) in low-resolution SDS-agarose gels in patients with type 2A/2M VWD and some discrepancy. VWF from platelet lysate (NPt), plasmas of a normal subject (NP), patients with type 2A/2M VWD and a patient with VWD type 2A (IIA) used as a control 2A are shown. **(a-c)** Patients that showed discrepancy between ratios and multimeric analysis. In the case of the patients C30P012F07, C30P013F08 and C37P003F03 the mutations had not been described previously and the multimeric analysis (smeary) was of great significance to establish the diagnosis.

A total of 34 patients from 13 families were classified as type 2A/2M VWD in this registry. Considering only the phenotypic study, the MA was of great significance because the “smear” multimeric pattern is distinctive of this type of VWD. In 21 out of 34 patients there were inconsistencies between ratios/MA **(S6 Table)**. Once the genetic study was completed, four mutations were found: p.Arg1374Cys (22 patients), p.Arg1315Cys (seven patients), p.Cys2491Arg (two patients) and p.Arg763Ser (three patients). The latter mutation was not previously described and the p.Cys2491Arg mutation had been described previously as type 3, in these two cases the multimeric study was again of great significance to establish the diagnosis **(Fig 5)**.

#### Type 3 VWD carriers

In this project, a total of 26 type 3 VWD carriers were included. Eight of them presented with some kind of discrepancy. Prior to genetic study, two patients had normal MA but both ratios were altered **(S7 Table and S6 Fig)**. After molecular analysis, it could be ascertained that both carried known mutations characteristic of type 3 VWD when they were in homozygous state. In the remaining six patients the phenotypic study was not in line with the genetic study. According to phenotypic study, the six would have been diagnosed as type 2M but *VWF* sequencing identified mutations previously described as causing type 3 VWD when they were in homozygous state **(S7 Table).**

MA served as confirmatory test of the other phenotypic tests and of the genetic study in these patients as well as in type 2N, type 2N carriers and type 3 VWD.

## Discussion

Despite that the latest official classification of VWD refers to the VWF multimers profile **[12]**, including the MA as an integral part of the diagnostic process, the role of MA of VWF in the diagnosis of VWD remains a matter of controversy due to its complexity, being considered by some clinicians as not very informative. Additionally, it is considered that a proper diagnosis includes RIPA, however, this assay is not available in all the laboratories and cannot be centralized, being a problem not only in Spain **[11].**

A preliminary report was recently released of the currently on-going External Quality Control of Diagnostic Assays and Test (ECAT) on the cross-EQUA VWF testing study involving over 500 laboratories world-wide and included the MA. The results this study highlights the importance of MA for the right interpretation of a VWF defect (Favaloro et al, 2017).

Taking into account the 480 patients centrally diagnosed in the Spanish PCM-EVW-ES project **[3, 18],** and as one step further, the present study intended to analyse: 1) the contribution of MA in the diagnosis of VWD and 2) the value of the VWF MA in the discrepancies with regards to VWF:RCo/VWF:Ag, VWF:CB/VWF:Ag and/or the genetic analysis.

Regarding the contribution of MA in this study, this test was of great significance in the diagnosis of 83 patients (17.3%). However, it was of help or in agreement in the diagnosis of 365 patients (76%) (diagnosis achieved in other steps). In consequence the MA global contribution (great significance plus concordant) was of 93.3% (448 patients) **(Fig 1).**

When the contribution of MA to the diagnosis was compared to that of VWF:CB **(Fig 2)** the data showed that the MA was clearly more efficient than the VWF:CB, which seems to reinforce the use of MA, although in case of no availability of the latter the former is useful.

With regard to discrepancies, 110 out of 480 (23%) patients centrally diagnosed with VWD presented some kind of discrepance between VWF:RCo/VWF:Ag, VWF:CB/VWF:CB, multimeric study and/or genetic analysis. In 78 out of 110 the discrepancies were between phenotypic parameters being the remaining 32 between phenotypic-genotypic studies.

As far as the molecular analysis is concerned, it was crucial in the distinction of types 2A/2B VWD cases, because MA is not informative to distinguish between both types and RIPA is not available in many centres.

As far as type 1 is concerned **(S2 Table)**, 24 out of 110 (21.8%) patients with discrepancy were type 1 VWD. Three of these patients carried the p.Pro1824His mutation that had been previously described first as type 1 **[25]** and later as type 2M **[26]**. In this project, these patients were classified as type 1 smeary since all of them presented a pronounced smearing pattern and they had increased FVIII:C/VWF:Ag ratios to around or above 2 **[29]**. In three other unrelated patients (C02P001F01, C13P006F04 and C32P004F04) **both ratios** were diminished but with a normal MA and if the latter assay had not been carried out they would have been classified as type 2A or 2B when really, they were type 1. **From** a clinical point of view, this would be very important because such patients should be treated in a different way.

In the remaining 18 out of 24 **type 1** pati**ents** according to the ratios would be type 2M but here, the MA would not be of help because in both cases, type 1 and type 2M, the multimeric pattern is normal. Twenty-two out of 24 patients with type 1 with discrepancies presented with a known mutation and in all cases the mutation and MA were consistent.

Regarding type 2A VWD, the MA helped to establish the diagnosis in 16 patients. It would have been decisive if the genetic study had not been carried out, or in cases with novel mutations **(S3 Table)**. All these patients would have been classified as type 2M VWD, when really, they would be type 2A VWD. A few years ago, it was suggested that probably types 2A and 2M should not be distinguished **[8]**.

On the other hand, in 18 patients diagnosed finally as type 2A the genetic study was necessary for a correct classification because all of them presented with a normal multimeric pattern inconsistent with this subtype of disease. In two patients, both the ratios and multimeric pattern were normal, but the mutation has been previously described as causing type 2A **[30]**. Several causes could explain the normal multimeric pattern in these patients: 1) Blood samples were drawn when the patient was on treatment which was ruled out by the physician responsible for the patient; 2) The normal multimeric pattern could be characteristic for the p.Arg1374His mutation but this hypothesis was rejected because in this project 26 patients carried this mutation and 10 of them presented absence of HMWM and also this mutation had been described previously as causing abnormal MA pattern. After ruling out both hypothesis, the only explanation for this situation would be that the mutation could be associated to some phenotypic heterogeneity **[28,31,32]**.

Regarding type 2B, the MA was very important in the five patients who presented with a problem with their classification **(S4 Table)**. Considering only the ratios, and if the genetic study had not been carried out or the mutation would have not been known, four out of 5 patients would have been diagnosed as type 2M VWD and the other one as type 1 VWD. It is important to comment that in patients type 2B Mälmo, the genetic study was essential because the patients might be diagnosed erroneously as type 1 since MA in both types are similar. Regarding the RIPA unavailability in many centers, not being possible the centralization of this assay, MA does not solve the problem in the identification of types 2A and 2B VWD. The more appropriate way to solve it is by making genetic analysis. It is worthy of mention that in type 2B, it has been recently reported the multimeric profile may be useful in distinguishing those patients with a presence of HMWM VWF in which DDAVP might be employed **[33]**.

Regarding type 2M, the establishment of a definitive diagnostic was difficult in eighteen out of 39 patients of this project. In 13 of them, while the MA was normal, both ratios were diminished and, according to the latest data, they should be classified as type 2A or type 2B VWD **(S5 Table)**. In the remaining five patients, the MA and the ratios were normal indicating type 1 VWD but they carried mutations previously described as type 2M. In all these case, the genetic analysis was very important being of great significance in the patients who carried mutations that affected to the binding of VWF to collagen VI (i.e. p.Arg1399His) since the test which evaluate this function is not included in routine laboratory assessment, and the patients with this mutation may have clinically severe type 2M VWD and may benefit from VWF replacement therapy for bleeding **[27]**. In this study, we carried out this assay in all patients who presented this type of mutations and in all of them it was confirmed a severe decrease in this activity **(S5 Table).**

With respect to type 2A/2M VWD, due to its characteristic multimeric distribution, the MA would have been of great significance in the 34 patients diagnosed in this registry if the genetic study had not been carried out (not only in the 21 patients with discordance) **(S6 Table)**.

After carrying out the *VWF* analysis, this assay was key in five patients because they carried mutations not described in EAHAD-VWD database (p.Cys2491Arg, p.Arg763Ser).

Last, but not the least, eight patients whom finally turned out to be type 3 carrier **(S7 Table)**, if only the ratios were considered two of them would be classified as type 2A/2B and the remaining six as type 2M. In all of cases the mutations were known, and they could be classified correctly. Although the MA may be seen unnecessary in type 3 VWD, because the absence of VWF, it was helpful in the present project detecting some problem, like a sampling error in a very few cases, and in confirming the lack of VWF in the remaining patients.

Regarding to potential methodological problems they were solved by retesting or analyzing new samples. In addition, the presence of unjustified discrepancies or not well understood diagnosis were discussed between central and local laboratories to identify potential methodological problems. We are aware that despite the best standardization possible, some problems may be overlooked.

## Conclusion

At present, the achievement of an accurate and precise diagnostic definition seems to require a set of different and complementary assays, including VWF MA. Discrepancies between either VWF:Ag and VWF:RCo or VWF:Ag and VWF:CB were more frequently than expected in the present study, complicating the diagnosis orientation. Detailed MA helped to make a more appropriate diagnosis assignment. In conclusion, despite the controversy raised by some investigators, data from the present study seem to support a sufficiently important role of the MA, reinforcing the value of this methodology in VWD laboratory workup, as stipulated by the ISTH revised classification **[12]**. Due to limitations that some centres present in carrying out the genetic analysis, the multimeric study would be of a great importance on many occasions. The complexity and/or difficulty in performing this test could be resolved with the centralisation in laboratories that have the necessary experience to achieve the best results. Although, it is becoming evident a valuable increasing role of *VWF* molecular analysis, contributing to a more precise diagnosis, it should not be considered as a surrogate test of the VWF phenotypic analysis. Rather, both the latter and the former should be complementary with the aim of achieving the most accurate diagnosis possible.

## Supporting information

S1 Table Modifications to the VWD revised ISTH classification. The following categories have been added in the PCM-EVW-ES project.
(PDF)

S2 Table Patients Type 1 VWD included in the PCM-EVW-ES who present some kind of discrepancy. (PDF)

S3 Table Patients with type 2A VWD who present some kind of discordancy. (PDF)

S4 Table Patients Type 2B with discordance between VWF:RCo/VWF:Ag-VWF:CB/VWF:Ag-multimeric pattern. (PDF)

S5 Table Patients with type 2M VWD who present some kind of discordancy. (PDF)

S6 Table Patients type 2A/2M with discordance between VWF:RCo/VWF:Ag-VWF:CB/VWF:Ag-multimeric pattern. (PDF)

S7 Table Type 3 VWD carriers included in the PCM-EVW-ES who present some kind of discrepancy. (PDF)

S1 Fig Schematic representation that highlight the discrepancy between ratios *versus* MA or ratios in concordance with MA, but discrepant with molecular analysis. A) Distribution in all cohort of 480 patients included in the PCM-EVW-ES. In total 110 patients present a discrepancy; B) Distribution by VWD type. MA: Multimeric analysis.
(TIF)

S2 Fig Multimeric analysis of von Willebrand factor (VWF) in low-resolution SDS-agarose gels in patients with type 2A VWD and some discrepancy. VWF from platelet lysate (NPt), plasmas of a normal subject (NP), patients with type 2A VWD and a patient with VWD type 2A (IIA) used as a control 2A are shown. All patients presented discordance between ratios and MA. In these cases, MA resulted difficult to define, in a first moment it was considered as “not conclusive”, but due to its similitary with the normal pattern finally were considered to be normal. The molecular study determined these patients carried p.Arg1374Cys mutation previously described as responsible of type 2A VWD.
(TIF)

S3 Fig Multimeric analysis of von Willebrand factor (VWF) in low-resolution SDS-agarose gels in patients with type 1 VWD and some discrepancy. VWF from platelet lysate (NPt), plasmas of a normal subject (NP), patients with type 1 VWD and a patient with VWD type 2A (IIA) used as a control 2A are shown. All patients presented discordance between ratios and MA. According to ratios they would be diagnosed as type 2A or 2B VWD, but according to MA they would be classified as type 1 (C02P001F01, C13P006F04, C37P005F05 and C39P019F08) or type 1 smeary (C02P047F18).
(TIF)

S4 Fig Multimeric analysis of von Willebrand factor (VWF) in low-resolution SDS-agarose gels in patients with type 2B VWD and some discrepancy. VWF from platelet lysate (NPt), plasmas of a normal subject (NP), patients with type 2B VWD and a patient with VWD type 2A (IIA) used as a control 2A are shown. All patients presented discordance between ratios and MA. All of them had known mutation and this mutation always was in line with the MA **(a-e)**.
(TIF)

S5 Fig Multimeric analysis of von Willebrand factor (VWF) in low-resolution SDS-agarose gels in patients with type 2M VWD and some discrepancy. VWF from platelet lysate (NPt), plasmas of a normal subject (NP), patients with type 2M VWD and a patient with VWD type 2A (IIA) used as a control 2A are shown. All of them presented discrepancy between ratios and MA. The patients C44P003F02 and C01P066F032 carried the mutation p.Gly1415Asp, previously described as type 1, but showing a phenotype compatible with type 2M in this registry **(a-b)**. Other patients presented mutation did not previously described but considered type 2M due to the presence of normal MA and VWF:RCo/VWF:Ag ratio diminished **(b-c)**.
(TIF)

S6 Fig Multimeric analysis of von Willebrand factor (VWF) in low-resolution SDS-agarose gels in type 3 carriers and some discrepancy. VWF from platelet lysate (NPt), plasmas of a normal subject (NP), type 3 carriers and a patient with VWD type 2A (IIA) used as a control 2A are shown. All patients presented discordance between ratios and MA. All of them had normal MA but the other parameters phenotypic were not in line with a type 3 carrier diagnosis **(a, b, c)**.
(TIF)
