# Supplementary material for: Role of multimeric analysis of von Willebrand factor (VWF) in von Willebrand disease (VWD) diagnosis: Lessons from the PCM-EVW-ES Spanish project
Source: PLoS One. 2018 Jun 20;13(6):e0197876. doi: 10.1371/journal.pone.0197876 (PMC6010290; doi:10.1371/journal.pone.0197876)
Supplement: S1 Table — The following categories have been added in the PCM-EVW-ES project. (PDF) [file pone.0197876.s001.pdf]

**Type 1H (1 “Historical”)**
Type 1 diagnosis clear in the past and currently does not show VWF levels ≤30% but has current bleeding symptoms. Most of these patients show some *VWF* mutation.
**VWD 1 (“Smeary”)**
Type 1 with smearing profile of all the multimers.
**VWD 2A/2M**
VWD that has been a matter of controversy receiving several different denominations showing multimeric smearing, with a variable proportion of high molecular weight multimers (HMWM). It is the case with p.Arg1374Cys and p.Arg1315Cys VWD.

VWF: von Willebrand factor; VWD: von Willebrand disease
