# Supplementary material for: Role of multimeric analysis of von Willebrand factor (VWF) in von Willebrand disease (VWD) diagnosis: Lessons from the PCM-EVW-ES Spanish project
Source: PLoS One. 2018 Jun 20;13(6):e0197876. doi: 10.1371/journal.pone.0197876 (PMC6010290; doi:10.1371/journal.pone.0197876)
Supplement: S2 Table — (PDF) [file pone.0197876.s002.pdf]

S2. Table. Patients Type 1 VWD included in the PCM-EVW-ES who present some kind of discrepancy.

Patient	FVIII:C (IU/dL)	VWF:Ag (IU/dL)	VWF:Rco (IU/dL)	VWF:CB (IU/dL)	VWF:RCo/ VWF:Ag	VWF:CB/ VWF:Ag	Multimeric analysis	Mutation	Type
C02P001F01	19	9.7	2.6	6	0.27	0.62	Normal¶	**p.Arg1205His***	1
C13P006F04	16	10	6	6.1	0.6	0.61	Normal¶	**p.Arg1205His***	1
C32P004F04	6.3	9.8	4	5	0.41	0.51	Normal¶	c.2821-123A>C	1
C02P047F18	30	9.3	6.4	6.1	0.69	0.66	Smear¶	p.Pro1824His*	1 Smeary
C02P049F18	37	11	6.6	8	0.6	0.73	Smear	p.Pro1824His*	1 Smeary
C02P050F18	37	10	6.9	7.1	0.69	0.71	Smear	p.Pro1824His*	1 Smeary
C02P016F01	34	16	9	16	0.56	1	Normal§	p.Arg1205His*	1
C13P012F09	20	14	9	11	0.64	0.79	Normal§	p.Arg1205His*	1
C14P003F03	61	28	20	18	0.71	0.64	Normal§	p.Gly160Arg*	1
C18P001F01	17	10	6.1	8.2	0.61	0.82	Normal§	p.Arg1205His/p.Val2330Gly*	1
C18P002F01	16	12	7	9.5	0.58	0.79	Normal§	p.Arg1205His/p.Val2330Gly*	1
C36P008F06	37	34	14	25	0.41	0.73	Normal§	p.Arg924Gln/c.3390C>T(p.=)*	1
C38P010F05	86	43	27.5	36	0.64	0.84	Normal§	p.Arg960Trp*	1
C45P001F01	16	11	6.6	9.4	0.6	0.85	Normal§	p.Arg1205His*	1
C37P005F05	44	26	18	18	0.69	0.69	Normal¶	p.Arg960Trp*	1
C39P017F08	44	26	17	19	0.65	0.73	Normal§	c.3390C>T*	1
C39P019F08	30	23	12	16	0.52	0.69	Normal¶	c.3390C>T*	1
C42P003F03	50	60	48	41	0.8	0.68	Normal§	p.Tyr1584Cys/p.Arg1916Gln*	1
C12P018F11	51	63	49	42	0.78	0.67	Normal§	p.Arg1583Trp*	1H
C19P006F04	74	46	38	31	0.83	0.67	Normal§	p.Gln2470Ter*	1H
C21P019F17	59	57	50	39	0.88	0.68	Normal§	p.Arg924Gln*	1H
C29P004F03	42	59	48	41	0.81	0.66	Normal§	p.Val1760Ile*	1H
C39P018F08	69	53	43	35	0.81	0.66	Normal§	c.3390C>T(p.=)*	1H

C32P007F07	46	69	35	34	0.51	0.49	Normal¶	p.Ile482Met	1H
NV	60-140	47-190	50-170	60-130	>0.7	>0.7	–	–	–

NV: Normal value; FVIII:C: procoagulant factor VIII; VWF:Ag: VWF antigen; VWF:RCo: VWF ristocetin cofactor activity; VWF:CB: VWF collagen binding.

Mutations previously described are indicated in bold type.

* Multimeric pattern consistent with the mutation.

¶ Discordance between ratios and multimeric pattern.

§ Ratios and multimeric structure matched but without coincidence with the genetic study.
