# Supplementary material for: Role of multimeric analysis of von Willebrand factor (VWF) in von Willebrand disease (VWD) diagnosis: Lessons from the PCM-EVW-ES Spanish project
Source: PLoS One. 2018 Jun 20;13(6):e0197876. doi: 10.1371/journal.pone.0197876 (PMC6010290; doi:10.1371/journal.pone.0197876)
Supplement: S3 Table — (PDF) [file pone.0197876.s003.pdf]

S3 Table. Patients with type 2A VWD who present some kind of discordancy.

Patient	FVIII:C (IU/dL)	VWF:Ag (IU/dL)	VWF:Rco (IU/dL)	VWF:CB (IU/dL)	VWF:RCo/ VWF:Ag	VWF:CB/ VWF:Ag	Multimeric analysis	Mutation	Type
C03P007F04	30	18	6.4	15	0.36	0.83	↓HMWM¶	**p.Arg1374His***	2A
C03P008F04	26	14	7	14	0.5	1	↓HMWM¶	**p.Arg1374His***	2A
C30P005F04	26	22	5.8	15	0.26	0.68	Normal¶	**p.Arg1374His**	2A
C30P015F04	29	31	5	14	0.16	0.45	Normal¶	**p.Arg1374His**	2A
C30P016F04	26	22	5	12	0.23	0.54	Normal¶	**p.Arg1374His**	2A
C30P019F04	21	26	5	12	0.19	0.46	Normal¶	**p.Arg1374His**	2A
C30P020F04	45	62	5.6	27	0.09	0.43	Normal¶	**p.Arg1374His**	2A
C30P021F04	33	35	5.8	17	0.19	0.49	Normal¶	**p.Arg1374His**	2A
C30P022F04	25	30	5.6	14	0.19	0.47	Normal¶	**p.Arg1374His**	2A
C30P024F04	19	25	5.4	14	0.22	0.56	Normal¶	**p.Arg1374His**	2A
C30P025F04	25	26	4	11	0.15	0.42	Normal¶	**p.Arg1374His**	2A
C30P026F04	27	33	4	18	0.12	0.55	Normal¶	**p.Arg1374His**	2A
C30P031F04	21	22	4	11	0.18	0.5	Normal¶	**p.Arg1374His**	2A
C30P032F04	41	57	9	29	0.16	0.51	Normal¶	**p.Arg1374His**	2A
C30P034F04	21	21	5	9.8	0.24	0.47	Normal¶	**p.Arg1374His**	2A
C30P036F04	30	23	5	11	0.22	0.48	Normal¶	**p.Arg1374His**	2A
C39P005F03	26	13	7.1	14	0.55	1.08	↓HMWM¶	**p.Arg1374His***	2A
C39P006F03	23	12	4.7	14	0.39	1.17	↓HMWM¶	**p.Arg1374His***	2A
C06P021F15	31	13	5.7	13	0.44	1	↓HMWM¶	**p.Arg1374His***	2A
C06P022F15	30	16	6.1	16	0.38	1	↓HMWM¶	**p.Arg1374His***	2A
C39P001F02	35	18	5	17	0.28	0.94	↓HMWM¶	**p.Arg1374His***	2A
C39P004F02	29	13	6.7	15	0.52	1.15	↓HMWM¶	**p.Arg1374His***	2A
C39P011F06	31	17	7.7	21	0.45	1.23	↓HMWM¶	**p.Ser1506Leu***	2A
C39P012F06	13	8.2	5	6.9	0.61	0.84	↓HMWM¶	**p.Ser1506Leu***	2A

C42P010F10	52	69	58.5	45	0.85	0.65	↓HMWM¶	**p.Arg1597Gln***	2A
C22P005F05	47	12	6.9	11	0.58	0.92	↓HMWM¶	p.Val1414Gly	2A
C07P006F02	26	17	4	12	0.24	0.71	↓HMWM¶	p.Asp1614Asn	2A
C02P071F27	67	27	23.6	36	0.87	1.33	Normal§	**p.Arg976Cys/p.Pro2063Ser**	2A
C02P072F27	114	45	46	57	1.02	1.27	Normal§	**p.Arg976Cys/p.Pro2063Ser**	2A
C30P011F04	26	18	5.8	15	0.32	0.83	Normal§	**p.Arg1374His**	2A
C30P029F04	19	19	5.7	13	0.3	0.72	Normal§	**p.Arg1374His**	2A
C30P004F03	26	16	5.4	13	0.34	0.81	↓HMWM¶	**p.Leu1307Pro***	2A
C30P007F03	24	13	5.9	9.9	0.45	0.76	↓HMWM¶	**p.Leu1307Pro***	2A
C30P010F03	21	11	5.6	9.2	0.51	0.83	↓HMWM¶	**p.Leu1307Pro***	2A
NV	60-140	47-190	50-170	60-130	>0.7	>0.7	–	–	–

NV: Normal FVIII:C: procoagulant factor VIII; VWF:Ag: VWF antigen; VWF:RCo: VWF ristocetin cofactor activity; VWF:CB: VWF collagen binding; ↓HMWM: decreased proportion of high molecular weight multimers.

Mutations previously described are indicated in bold type.

* Multimeric pattern consistent with the mutation.

¶ Discordance between ratios and multimeric pattern.

§ Ratios and multimeric structure matched but without coincidence with the genetic study.
