# Supplementary material for: Role of multimeric analysis of von Willebrand factor (VWF) in von Willebrand disease (VWD) diagnosis: Lessons from the PCM-EVW-ES Spanish project
Source: PLoS One. 2018 Jun 20;13(6):e0197876. doi: 10.1371/journal.pone.0197876 (PMC6010290; doi:10.1371/journal.pone.0197876)
Supplement: S4 Table — (PDF) [file pone.0197876.s004.pdf]

S4 Table. Patients Type 2B with discordance between VWF:RCo/VWF:Ag-VWF:CB/VWF:Ag-multimeric pattern.

Patient	Platelet count (x10^{9}L)	FVIII:C (IU/dL)	VWF:Ag (IU/dL)	VWF:Rco (IU/dL)	VWF:CB (IU/dL)	VWF:RCo/ VWF:Ag	VWF:CB/ VWF:Ag	Multimeric analysis	Mutation	Type
C02P078F07	–	55	34	24	21	0.71	0.62	↓HMWM¶	**p.Arg1308Cys***	2B
C27P015F08	98	63	33	18.5	25	0.56	0.76	↓HMWM¶	**p.Arg1306Trp***	2B
C14P001F01	116	15	9	6	7.6	0.67	0.84	↓HMWM¶	**p.Arg1306Trp***	2B
C12P015F08	119	48	56	45	42	0.8	0.75	↓HMWM¶	**p.Arg1306Gln***	2B
C35P009F04	141	24	23	18	16	0.78	0.69	↓HMWM¶	**p.Arg1308Cys***	2B
NV	150-400	60-140	47-190	50-170	60-130	>0.7	>0.7	–	–	–

NV: Normal value; FVIII:C: procoagulant factor VIII; VWF:Ag: VWF antigen; VWF:RCo: VWF ristocetin cofactor activity; VWF:CB: VWF collagen binding; ↓HMWM: decreased proportion of high molecular weight multimers.

Mutations previously described are indicated in bold type.

* Multimeric pattern consistent with the mutation.

¶ Discordance between ratios and multimeric pattern.
