# Supplementary material for: Role of multimeric analysis of von Willebrand factor (VWF) in von Willebrand disease (VWD) diagnosis: Lessons from the PCM-EVW-ES Spanish project
Source: PLoS One. 2018 Jun 20;13(6):e0197876. doi: 10.1371/journal.pone.0197876 (PMC6010290; doi:10.1371/journal.pone.0197876)
Supplement: S5 Table — (PDF) [file pone.0197876.s005.pdf]

S5 Table. Patients with type 2M VWD who present some kind of discordancy

Patient	FVIII:C (IU/dL)	VWF:Ag (IU/dL)	WF:Rco (IU/dL)	VWF:CB (IU/dL)	VWF:^{VI}CB	VWF:RCo/ VWF:Ag	VWF:CB/ VWF:Ag	Multimeric analysis	Mutation	Type
C01P066F32	26	16	4	11		0.25	0.69	Normal¶	**p.Gly1415Asp***	2M
C12P020F13	67	84	55	53	4.59	0.65	0.63	Normal¶	**p.Arg1399His***	2M
C27P010F06	18	6.8	4	4.3		0.59	0.63	Normal¶	**p.Leu1382Pro***	2M
C03P023F32	21	12	4.4	7.7		0.37	0.64	Normal¶	p.Val1409Phe	2M
C03P024F32	26	25	7.2	12	0.1	0.29	0.48	Normal¶	p.Val1409Phe/**p.Arg1399His***	2M
C03P025F32	65	109	17	42		0.16	0.38	Normal¶	p.Val1409Phe	2M
C12P023F16	213	272	186	178		0.68	0.65	Normal¶	p.Arg1395Trp	2M
C30P027F10	34	36	16	20		0.44	0.55	Normal¶	p.Arg1779Leu	2M
C30P028F10	19	28	13.6	16		0.49	0.57	Normal¶	p.Arg1779Leu	2M
C44P003F02	11	16	5	11		0.31	0.69	Normal	**p.Gly1415Asp/Arg854Gln***	2M
C44P008F06	23	28	6.2	19		0.22	0.68	Normal¶	p.Val1409Phe	2M
C44P009F06	60	46	10.4	32		0.23	0.69	Normal¶	p.Val1409Phe	2M
C44P010F06	33	33	7.4	21		0.22	0.64	Normal¶	p.Val1409Phe	2M
C01P036F18	17	16	24	20	1.48	1.5	1.25	Normal	**p.Arg1399His***	2M
C05P013F12	78	50	47.6	39	6.14	0.95	0.78	Normal	**p.Arg1399His***	2M
C07P007F05	97	32	39	24	0	1.22	0.75	Normal	**p.Arg1399His**/p.Cys2283Arg*	2M
C27P009F06	15	5.4	4	5.6		0.74	1.03	Normal	**p.Leu1382Pro***	2M
C27P022F12	65	38	35.5	41	0.11	0.93	1.08	Normal	**p.Ser1731Thr***	2M
NV	60-140	47-190	50-170	60-130	60-130	>0.7	>0.7	–	–	–

NV: Normal value; FVIII:C: procoagulant factor VIII; VWF:Ag: VWF antigen; VWF:RCo: VWF ristocetin cofactor activity; VWF:CB: VWF collagen binding.

Mutations previously described are indicated in bold type.

* Multimeric pattern consistent with the mutation.

¶ Discordance between ratios and multimeric pattern.
