# Supplementary material for: Role of multimeric analysis of von Willebrand factor (VWF) in von Willebrand disease (VWD) diagnosis: Lessons from the PCM-EVW-ES Spanish project
Source: PLoS One. 2018 Jun 20;13(6):e0197876. doi: 10.1371/journal.pone.0197876 (PMC6010290; doi:10.1371/journal.pone.0197876)
Supplement: S6 Table — (PDF) [file pone.0197876.s006.pdf]

S6 Table. Patients type 2A/2M with discordance between VWF:RCo/VWF:Ag-VWF:CB/VWF:Ag-multimeric pattern

Patient	FVIII:C (IU/dL)	VWF:Ag (IU/dL)	VWF:Rco (IU/dL)	VWF:CB (IU/dL)	VWF:RCo/ VWF:Ag	VWF:CB/ VWF:Ag	Multimeric analysis	Mutation	Type
C02P026F08	44	21	7.4	8.8	0.35	0.42	*Smear*¶	**p.Arg1315Cys***	2A/2M
C02P027F08	28	11	7.1	6.4	0.64	0.58	*Smear*¶	**p.Arg1315Cys***	2A/2M
C02P034F12	23	26	8.4	14	0.32	0.54	*Smear*¶	**p.Arg1315Cys***	2A/2M
C03P017F76	39	44	11.8	23	0.27	0.52	*Smear*¶	**p.Arg1374Cys***	2A/2M
C13P009F07	20	9.5	5	5.8	0.53	0.61	*Smear*¶	**p.Arg1315Cys***	2A/2M
C13P016F07	21	11	5	6.7	0.45	0.61	*Smear*¶	**p.Arg1315Cys***	2A/2M
C01P038F20	20	20	5.3	13	0.27	0.65	*Smear*¶	**p.Arg1315Cys***	2A/2M
C01P002F02	34	16	4.4	9.2	0.28	0.58	*Smear*¶	**p.Arg1374Cys***	2A/2M
C01P003F02	45	20	6.7	13	0.34	0.65	*Smear*¶	**p.Arg1374Cys***	2A/2M
C01P020F02	47	32	11	19	0.34	0.59	*Smear*¶	**p.Arg1374Cys***	2A/2M
C01P022F02	59	54	18	30	0.33	0.55	*Smear*¶	**p.Arg1374Cys***	2A/2M
C01P039F02	24	23	12	15	0.52	0.65	*Smear*¶	**p.Arg1374Cys***	2A/2M
C01P007F04	38	25	7.8	15	0.31	0.6	*Smear*¶	**p.Arg1374Cys***	2A/2M
C01P015F04	36	19	7.8	11	0.41	0.58	*Smear*¶	**p.Arg1374Cys***	2A/2M
C01P023F04	27	30	13	18	0.43	0.6	*Smear*¶	**p.Arg1374Cys***	2A/2M
C01P033F04	32	27	10	16	0.37	0.59	*Smear*¶	**p.Arg1374Cys***	2A/2M
C01P042F16	26	27	4.9	17	0.18	0.63	*Smear*¶	**p.Arg1374Cys***	2A/2M
C01P071F16	17	13	6.6	6.7	0.51	0.51	*Smear*¶	**p.Arg1374Cys***	2A/2M
C37P003F03	44	30	25	20	0.83	0.66	*Smear*¶	p.Cys2491Arg	2A/2M
C30P012F07	18	32	28	19	0.88	0.59	*Smear*¶	p.Arg763Ser	2A/2M and 2N
C30P013F08	53	78	31.8	34	0.41	0.43	*Smear*¶	p.Arg763Ser	2A/2M and 2N
NV	60-140	47-190	50-170	60-130	>0.7	>0.7	–	–	–

NV: Normal value; FVIII:C: procoagulant factor VIII; VWF:Ag: VWF antigen; VWF:RCo: VWF ristocetin cofactor activity; VWF:CB: VWF collagen binding.

Mutations previously described are indicated in bold type.

* Multimeric pattern consistent with the mutation.

¶ Discordance between ratios and multimeric pattern.
