# Supplementary material for: Role of multimeric analysis of von Willebrand factor (VWF) in von Willebrand disease (VWD) diagnosis: Lessons from the PCM-EVW-ES Spanish project
Source: PLoS One. 2018 Jun 20;13(6):e0197876. doi: 10.1371/journal.pone.0197876 (PMC6010290; doi:10.1371/journal.pone.0197876)
Supplement: S7 Table — (PDF) [file pone.0197876.s007.pdf]

S7 Table. Type 3 VWD carrier included in the PCM-EVW-ES who present some kind of discrepancy

Patient	FVIII:C (IU/dL)	VWF:Ag (IU/dL)	VWF:Rco (IU/dL)	VWF:CB (IU/dL)	VWF:RCo/ VWF:Ag	VWF:CB/ VWF:Ag	Multimeric analysis	Mutation	Type
C03P022F25	85	88	46	48	0.52	0.55	Normal¶	**c.7082-2A>G*/c.7730-177G>T ***	3 carrier
C32P011F10	88	51	23.5	21	0.46	0.41	Normal¶	**p.Gln1311Ter***	3 carrier
C01P060F24	100	143	114	98	0.8	0.68	Normal	**p.Arg324Ter***	3 carrier
C03P038F127	82	67	37	56	0.55	0.84	Normal	**p.Gln1311Ter***	3 carrier
C03P039F127	68	66	34	47	0.52	0.71	Normal	**p.Gln1311Ter***	3 carrier
C27P041F21	66	42	25	48	0.59	1.14	Normal	**p.Tyr126Thrfster49***	3 carrier
C34P001F01	48	41	35	28	0.85	0.68	Normal	**p.Gly142Asp***	3 carrier
C39P010F05	128	104	92	57	0.88	0.55	Normal	**p.Gln2783Ter***	3 carrier
NV	60-140	47-190	50-170	60-130	>0.7	>0.7	–	–	–

NV: Normal value; FVIII:C: procoagulant factor VIII; VWF:Ag: VWF antigen; VWF:RCo: VWF ristocetin cofactor activity; VWF:CB: VWF collagen binding. Mutations previously described are indicated in bold type.

* Multimeric pattern consistent with the mutation.

¶ Discordance between ratios and multimeric pattern
