# Supplementary figures and images for: Role of multimeric analysis of von Willebrand factor (VWF) in von Willebrand disease (VWD) diagnosis: Lessons from the PCM-EVW-ES Spanish project
Source: PLoS One. 2018 Jun 20;13(6):e0197876. doi: 10.1371/journal.pone.0197876 (PMC6010290; doi:10.1371/journal.pone.0197876)

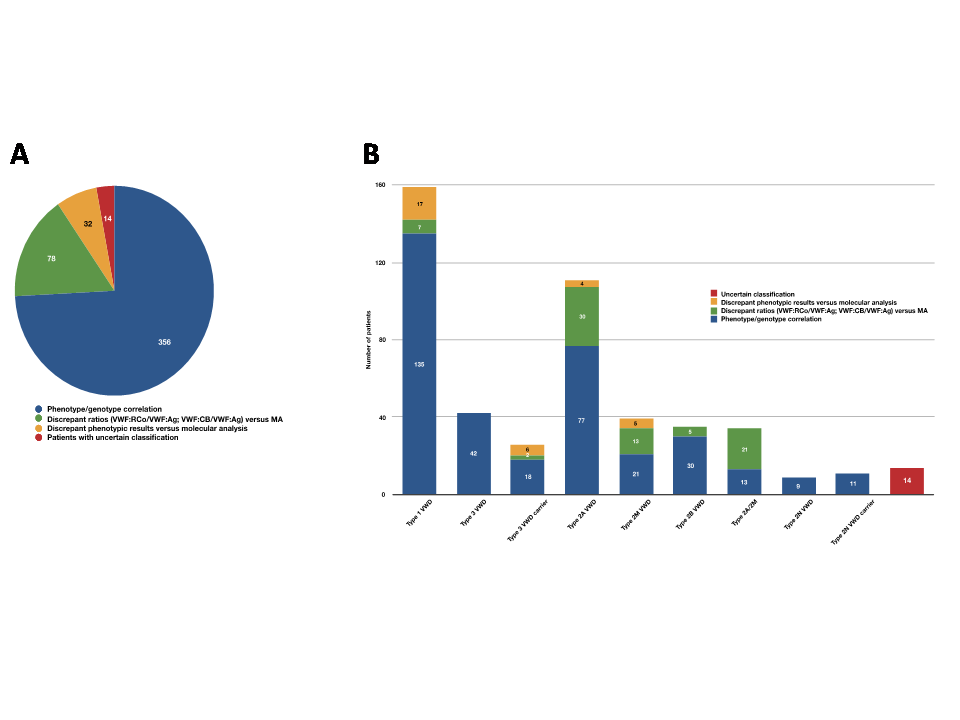

Supplement: S1 Fig — A) Distribution in all cohort of 480 patients included in the PCM-EVW-ES. In total 110 patients present a discrepancy; B) Distribution by VWD type. MA: Multimeric analysis. (TIF) [file pone.0197876.s008.tif]

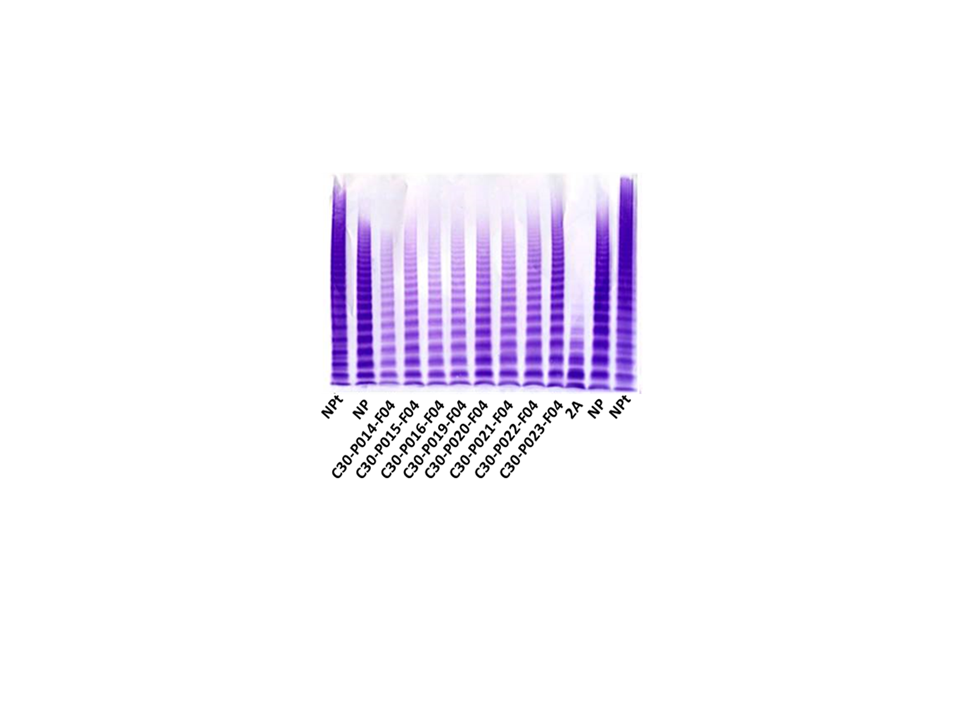

Supplement: S2 Fig — VWF from platelet lysate (NPt), plasmas of a normal subject (NP), patients with type 2A VWD and a patient with VWD type 2A (IIA) used as a control 2A are shown. All patients presented discordance between ratios and MA. In these cases, MA resulted difficult to define, in a first moment it was considered as “not conclusive”, but due to its similitary with the normal pattern finally were considered to be normal. The molecular study determined these patients carried p.Arg1374Cys mutation previously described as responsible of type 2A VWD. (TIF) [file pone.0197876.s009.tif]

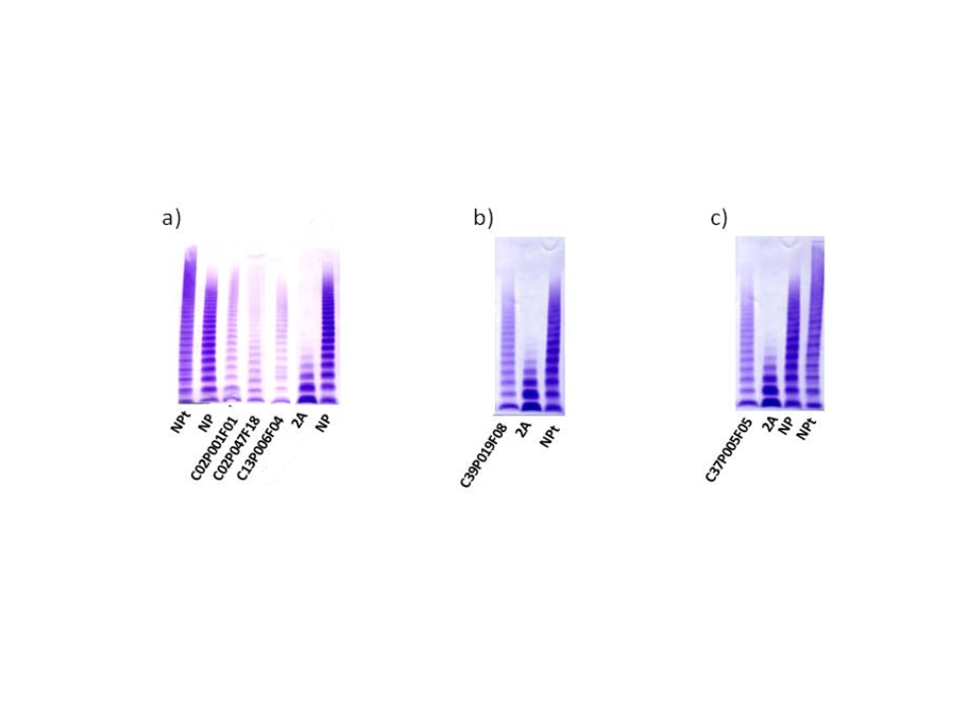

Supplement: S3 Fig — VWF from platelet lysate (NPt), plasmas of a normal subject (NP), patients with type 1 VWD and a patient with VWD type 2A (IIA) used as a control 2A are shown. All patients presented discordance between ratios and MA. According to ratios they would be diagnosed as type 2A or 2B VWD, but according to MA they would be classified as type 1 (C02P001F01, C13P006F04, C37P005F05 and C39P019F08) or type 1 smeary (C02P047F18). (TIF) [file pone.0197876.s010.tif]

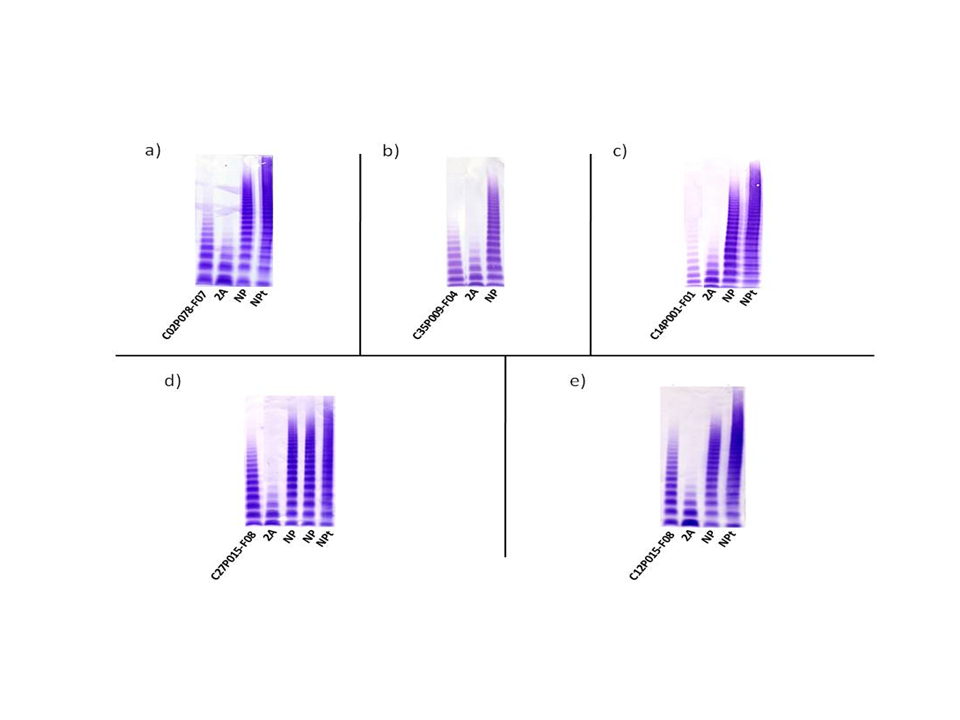

Supplement: S4 Fig — VWF from platelet lysate (NPt), plasmas of a normal subject (NP), patients with type 2B VWD and a patient with VWD type 2A (IIA) used as a control 2A are shown. All patients presented discordance between ratios and MA. All of them had known mutation and this mutation always was in line with the MA (a-e). (TIF) [file pone.0197876.s011.tif]

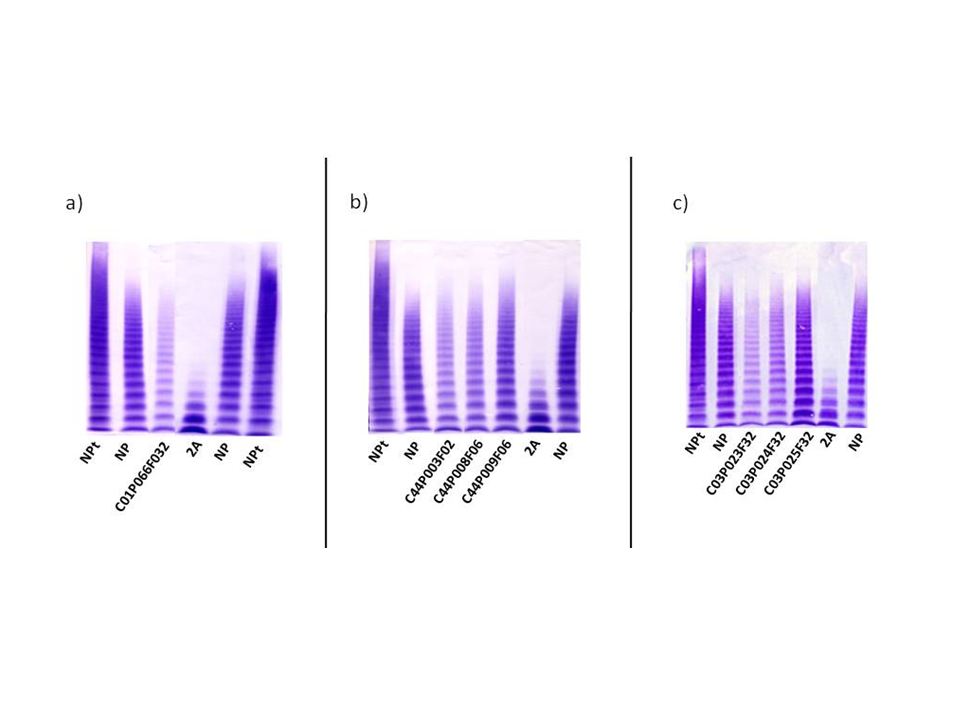

Supplement: S5 Fig — VWF from platelet lysate (NPt), plasmas of a normal subject (NP), patients with type 2M VWD and a patient with VWD type 2A (IIA) used as a control 2A are shown. All of them presented discrepancy between ratios and MA. The patients C44P003F02 and C01P066F032 carried the mutation p.Gly1415Asp, previously described as type 1, but showing a phenotype compatible with type 2M in this registry (a-b). Other patients presented mutation did not previously described but considered type 2M due to the presence of normal MA and VWF:RCo/VWF:Ag ratio diminished (b-c). (TIF) [file pone.0197876.s012.tif]

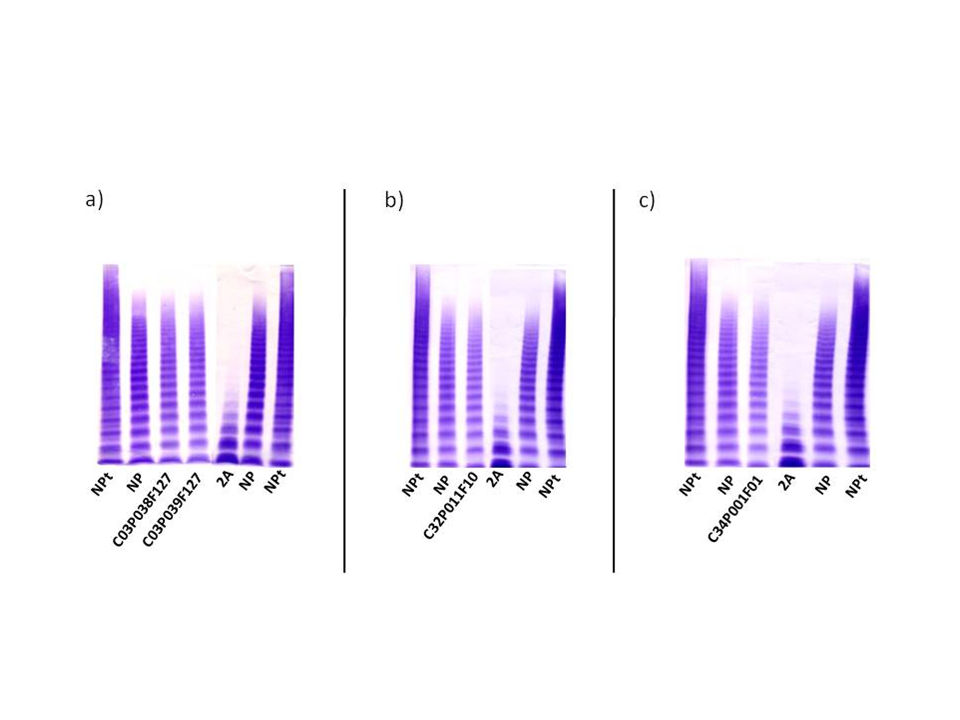

Supplement: S6 Fig — VWF from platelet lysate (NPt), plasmas of a normal subject (NP), type 3 carriers and a patient with VWD type 2A (IIA) used as a control 2A are shown. All patients presented discordance between ratios and MA. All of them had normal MA but the other parameters phenotypic were not in line with a type 3 carrier diagnosis (a, b, c). (TIF) [file pone.0197876.s013.tif]
